# Provisioning Mass by Females of the Maritime Earwig, *Anisolabis maritima*, is not Adjusted Based on the Number of Young

**DOI:** 10.1673/031.011.16001

**Published:** 2011-11-21

**Authors:** Seizi Suzuki

**Affiliations:** Department of Ecology and Systematics, Graduate School of Agriculture, Hokkaido University, Sapporo 060-8589, Japan

**Keywords:** begging signal, maternal care, parent—offspring conflict

## Abstract

The amount of parental provisioning is thought to reflect the need of offspring. This hypothesis was tested in the case of provisioning food mass to young with controlled clutch size using the maritime earwig, *Anisolabis maritima* Bonelli (Dermaptera: Anisolabididae). The female provisioned a constant mass of food to the young irrespective of the number of nymphs and the distance of food carrying. In addition, the survival rate of young did not change with adjusted clutch size. This study showed that *A. maritima* females appear to provide food mass to their nymphs independent of their number.

## Introduction

Provisioning has been reported for several insect species (Costa 2006). Progressive provisioning, when parents repeatedly transport food to their young, can be regarded as a special form of parental care that is found in some insect species (Filippi-Tsukamoto et al. 1995; Filippi et al. 2001). Parental care can only spread if the benefits of care outweigh the offspring number advantage of non—caring individuals. Food provisioning to the young by parents is a widespread phenomenon that enhances the survival of the young, but preparing the food can be very costly to the parents (Clutton-Brock 1991). Thus, the mechanism of food allocation to the young is an important issue of evolutionary biology. It is possible that some parents have a flexible investment dependent upon their young's need, in order to minimize the costs of current reproduction (Sæther et al. 1993).

The amount and duration of parental investment are also influenced by a conflict between parents and offspring for the need of investment (Trivers 1974). The evolution of offspring begging signals is predicted based on an evolutionary resolution of this conflict (Parker et al. 2002). Begging signals are thought to reflect the needs of offspring, which parents use to adjust their food allocation (Godfray 1991). Recently, the presence of food begging signals for parents has been recognized in some insect species (Kölliker et al. 2005; Smiseth and Moore 2004). All the species of earwig (Dermaptera) studied to date exhibit parental care (Lamb 1976), though the extent of care varies greatly from species to species (Vancassel 1984). Furthermore, the females of some species have been reported to provision their nymphs (Shepard et al. 1973; Lamb 1976; Rankin et al. 1996; Staerkle and Kölliker 2008). Mas et al. (2009) reported the presence of chemical begging signals to their mother, this group will be suitable material for examining the regulation of female—offspring interactions.

*Anisolabis maritima* Bonelli (Dermaptera: Anisolabididae) is a cosmopolitan earwig species that exhibits subsocial behavior, where the females tend clutches of eggs in soil burrows (Bennett 1904). The females provision nymphs progressively, and providing food to the nymphs increases the nymph survival (Suzuki 2010). Suzuki (2010) reported that *A. maritima* females provision increasing amounts of food as the number of days from hatching increases. Provisioning in *A. maritima* may play a crucial part in the maintenance of family groups, because the survival rate of nymphs decreased greatly in the absence of provisioning by the female (Suzuki 2010). Thus, it is possible that females control food mass or provisioning times for nymph needs. Since *A. maritima* females lack kin recognition ability (Suzuki, unpublished data), their clutch size can be manipulated easily, and the manipulation can be expected to cause a change in the intensity of begging signals by the young. The aim of this study was to determine, by controlling clutch size, whether *A. maritima* females regulate their food mass provisioning in response to their young's needs. The effect of the size of food mass on the survival of nymphs was also studied.

## Materials and Methods

All *A. maritima* earwigs were caught in a field at the coast of Izumozaki, Niigata prefecture, Japan (37° 32′ 11″ E, 138° 42′ 10″ N) from late April to early May in 2009. All females were paired with a male for more than one day prior to the start of the experiment. After measuring body length, females were placed in a polyethylene container (8 × 5 × 4 cm) on a 1–5 mm layer of moist sand. Containers were placed under dim light at room temperature with sufficient humidity. All individuals were fed with turtle food pellets *ad libitum*. All containers including females were checked daily, and when egg masses were found, the containers containing the masses were subjected to the experiments.

Experiment 1 (adjusted clutch size): The mean size of the typical *A. maritima* first clutch is 58.0 eggs, with a range of 19–125 (Suzuki, unpublished data). Two or more clutches were produced within two days, and eggs were moved carefully with forceps. Clutch size was adjusted to 30, 60, or 90 eggs (adjusted clutch size) after determining the original clutch size. Since *A. maritima* females cannot distinguish their own eggs from a stranger's eggs (Suzuki, pers. obs.), they treated the adjusted clutches normally. 16 clutches were adjusted as 30 eggs, 21 clutches as 60 eggs, and 16 clutches as 90 eggs. When hatched nymphs were found, food for nymphs was placed beside them. Bottle caps (25 mm diameter, 10 mm depth) placed at a distance of 2–3 cm from the burrow were used as food containers. Immature (before dispersal) nymphs could not enter the bottle cap and eat the food (Suzuki 2010). The food provided was 15 turtle food pellets (average 0.1 g), a mass that is 1.5 times or more than the need of nymphs in a normal—sized brood (Suzuki 2010). Each container was checked daily, at which time the uneaten pellets were counted and replenished by adding up to 15 pellets as needed. When more than half of the nymphs had left the nests, or some nymphs were present in the bottle cap, the brood was recorded as dispersed. Since the day of hatching cannot be synchronized within an adjusted clutch, the time span ranging from the first day of food carrying to dispersal day was regarded as the duration of care. After nymph dispersal, the number of surviving nymphs was counted.

Experiment 2 manipulated the distance between food source and nest to potential verify the cost of provisioning. After confirming egg mass, the females and eggs were placed in larger boxes (20 × 7 × 6 cm) and clutch sizes were adjusted to 60 (N = 11). Bottle caps (25mm diameter, 10mm depth) placed at a distance of 15 cm from the burrow were used as food containers. Food was provided for hatched nymphs once they were found. Using same procedure of Experiment 1, 15 turtle food pellets were provided. Each container was checked daily, at which time uneaten pellets were counted and replenished by adding up to 15 pellets as needed.

## Results

**Figure 1.**
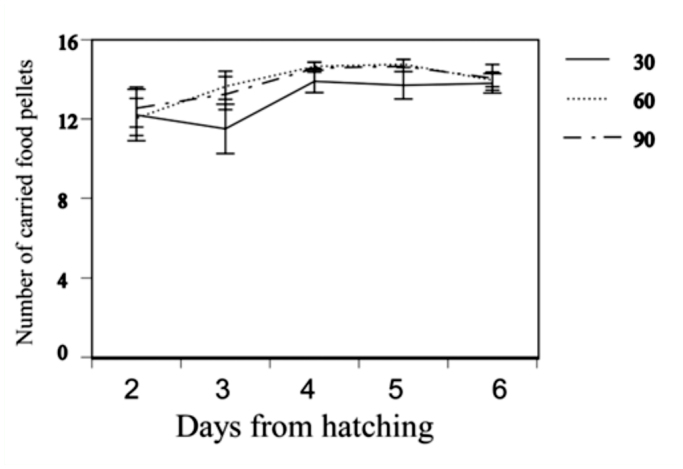
Daily changes and difference among adjusted clutch size in the average ± SE amount of food provisioning by *Anisolabis maritima.* High quality figures are available online.

Experiment 1 (adjusted clutch size): The original clutch size (before adjustment) did not differ among adjusted clutch size (30: 54.7 ± 9.3; 60: 60.9 ± 14.2; 90: 58.3 ± 15.9, one— way ANOVA, *F* = 0.91, df = 2, *p* = 0.4) and did not correlate with the body size of the adults (r = 0.19, *p* = 0.37). Thus, the effect of body size was excluded from the analysis. Despite adjusted clutch size, females carried most of the food from the bottle cap, and provisioning increased over time regardless of clutch size (Figure 1, GLM using REML analyses with individuals as a random factor, date: *F* = 15.9; df = 4, *p* < 0.01, clutch size difference: *F* = 1.71, df = 2, *p* = 0.12, date*clutch interaction: *F* = 0.14, *p* = 0.70). The survival rate of nymphs was not significantly different among broods of adjusted clutch size (one—way ANOVA after arcsine transformation, *F* = 0.6, df = 2, *p* = 0.9). Duration of care was also not significantly different among broods of adjusted clutch size (30: 4.9 ± 0.4; 60: 4.8 ± 0.4; 90: 4.9 ± 0.3, one—way ANOVA *F* = 0.16, df = 2, *p* = 0.8). However, since the day of hatching cannot be synchronized within an adjusted clutch, this duration of care seemed not to be exact.

**Figure 2.**
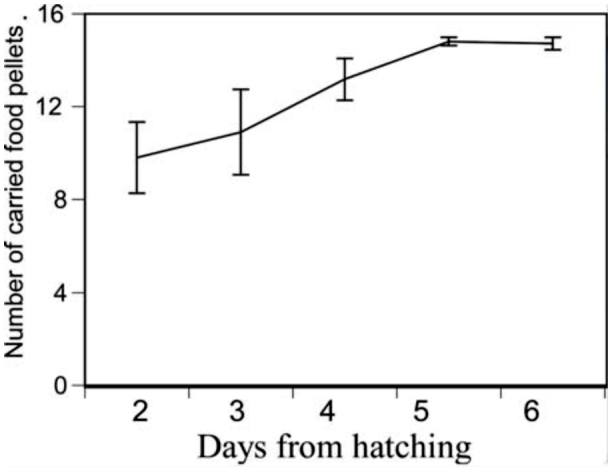
Daily changes in the average ± SE amount of food provisioning with increased distance from the food source by *Anisolabis maritima*. High quality figures are available online.

In Experiment 2, where the cost of care was manipulated by varying the distance to the food source, females still carried most of the food from the food cap regardless of the increased distance to the food source. Furthermore, the number of carried food pellets was not different from that found in Experiment 1 in which the distance to the food source was shorter (Repeated—measures ANOVA, *F* = 3.3, df = 1, *p* = 0.08, Figure 2).

## Discussion

Providing food to their offspring may cost parents not only because of higher risks of predation while foraging, but also increased energy expenditure. Parents reportedly gauge the young's need by measuring the level of offspring begging (Kilner and Johnstone 1997). Provisioning the young improves their survival rate but may decrease female energy intake required for future reproduction (Mas et al. 2008). However, the results of this experiment indicated that *A. maritima* females provision a constant mass of food to the young regardless of brood size, even though the survival rate of nymphs was independent of brood size, and females carried much more food than the young needed. Though provisioning improves the survival of young (Suzuki 2010), females of *A. maritima* will not control the mass of provision precisely.

In species where the parents perform food provisioning, reciprocal parent—offspring interaction drives the evolution of offspring begging signals. Begging signals are thought to reflect the hunger level of offspring, which parents use to adjust their food allocation (Godfray 1991). Empirical studies conducted on birds showed that feeding the offspring increased their begging (Kilner and Johnstone 1997). Food begging behavior by the young has been reported in some insect species (den Boer and Duchateau 2006; Kölliker et al. 2005, 2006; Smiseth et al. 2003). However, these studies have postulated both the presence of a begging signal and the ability of parents to recognize the begging signal. Unlike the burying beetle (Smiseth et al. 2003) or the Common European earwig (Staerkle and Kölliker 2008), *A. maritima* does not allocate food to individual young but brings food back for the whole brood (Suzuki 2010), similar to the burrower bug (Knight 1997). In such types of provisioning, the benefit of maternal food provisioning is simultaneously shared among all offspring, and competition among the young can disturb food allocation by the parents. Since begging signal has been reported in the nymphs of burrower bug (Kölliker et al. 2005), absence of individual food allocation will not be necessary to the evolution of the begging signal.

Progressive provisioning has been reported in some Cydnidae species (Filippi et al. 2001; Kölliker et al. 2006) that carry plant seeds as food for their young. In field conditions, *A. maritima* will eat small invertebrates (Bennett 1904; Suzuki, personal observation). Live and/or dead invertebrates are protein rich but seem to be difficult to collect constantly, although their availability in the field is unknown. As a resource this type of food can easily go rotten and is difficult to preserve. Foods for this experiment in the laboratory also easily go rotten.

The benefit of provisioning in *A. maritima* is substantial (Suzuki 2010), although the details of the cost have not been clarified. Since females seem to provide too much food for the young, females may pay an extra price for provisioning. The cost of care in some subsocial insects has been reported; for example, the interval from the first to the second clutch was shorter without care in the Dermapteran species *F. auricularia* (Vancassel and Foraste 1980; Vancassel 1984; Kölliker 2007) and *E. annulipes* (Rankin et al. 1996). Such delay may be common in Dermapteran species and may reduce the opportunity for reproduction. Agrawal et al. (2005) showed a very small cost of provisioning in the burrower bug (*Sehirus cinctus*). If the cost of provisioning food by *A. maritima* is negligibly small, it is unnecessary to minimize provision dependent upon the young's need. If this hypothesis is correct, females can provision in excess of their young's need, since provisioning more than is needed does not have a negative effect on reproduction. These experiments were planned to provide excess food for young's consumption (see Suzuki 2010). All experiments were conducted in artificial condition; the cost of provisioning may be lower in field conditions. There needs to be further investigation to verify this hypothesis.

In summary, this study asserts the possibility of the absence of controlling food provisioning in *A. maritima*. Although the current evolutionary theory on parent—offspring conflict resolution has generally assumed that offspring begging signals advertise need, this result showed the possibility that parents provide food in excess of need. Detailed behavioral experiments will be required to fully understand how to determine food provisioning to the young.
